# Electroacupuncture induces weight loss by regulating tuberous sclerosis complex 1-mammalian target of rapamycin methylation and hypothalamic autophagy in high-fat diet-induced obese rats

**DOI:** 10.3389/fphar.2022.1015784

**Published:** 2022-10-12

**Authors:** Junpeng Yao, Xiangyun Yan, Xianjun Xiao, Xi You, Yanqiu Li, Yuqing Yang, Wei Zhang, Ying Li

**Affiliations:** ^1^ Acupuncture and Tuina School/The 3rd Teaching Hospital, Chengdu University of Traditional Chinese Medicine, Chengdu, China; ^2^ School of Health Preservation and Rehabilitation, Chengdu University of Traditional Chinese Medicine, Chengdu, China; ^3^ Academic Affairs Office, Chengdu University of Traditional Chinese Medicine, Chengdu, China

**Keywords:** electroacupuncture, obesity, methylation, tuberous sclerosis complex 1, mammalian target of rapamycin, autophagy

## Abstract

**Background:** Obesity can be caused by abnormalities of hypothalamic autophagy, which is closely regulated by the epigenetic modification of TSC1-mTOR. However, whether the weight-reducing effect of EA may relate to the modification of TSC1-mTOR methylation and hypothalamic autophagy remain unclear. This study was conducted to reveal the possible mechanism by which EA reduces BW by measuring the levels of TSC1-mTOR methylation and hypothalamic autophagy-related components.

**Methods:** The weight-reducing effect of EA was investigated in high-fat diet (HFD)-induced obese (DIO) rats by monitoring the BW, food consumption, and epididymal white adipose tissue (eWAT)/BW ratio. Hematoxylin and eosin staining was performed for morphological evaluation of eWAT. Immunofluorescence was utilized to observe the localization of LC3 in the hypothalamus. The expressions of autophagy components (Beclin-1, LC3, and p62) and mTOR signaling (mTOR, p-mTOR, p70S6K, and p-p70S6K) were assessed by western blot. The methylation rate of the TSC1 promoter was detected by bisulfite genomic sequencing.

**Results:** Treatment with EA significantly reduced the BW, food consumption, and eWAT/BW ratio; attenuated the morphological alternations in the adipocytes of DIO rats. While HFD downregulated the expression levels of Beclin-1 and LC3 and upregulated those of p62, these changes were normalized by EA treatment. EA markedly decreased the methylation rate of the *TSC1* gene promoter and suppressed the protein expressions of mTOR, p-mTOR, p70S6K, and p-p70S6K in the hypothalamus.

**Conclusion:** EA could reduce BW and fat accumulation in DIO rats. This ameliorative effect of EA may be associated with its demethylation effect on TSC1-mTOR and regulation of autophagy in the hypothalamus.

## Introduction

Obesity results from an imbalance in energy intake and expenditure, the development of which is known to involve genetic factors. Excessive energy intake, insufficient exercise, and genetic susceptibility are the leading causes of obesity (Oussaada et al., 2019). Obesity is commonly seen even in some developing countries where child malnutrition is observed (Pan et al., 2021). According to the World Health Organization, the number of overweight people globally reached 1.9 billion in 2016, including 650 million obese people, and among adults (18 years and older), 39% and 13% were overweight and obese, respectively (Luo et al., 2018). As the prevalence of obesity increases, the prevalence of related chronic non-communicable diseases such as diabetes, hypertension, and cardiovascular and cerebrovascular diseases also significantly increases (Jehan et al., 2018). A health economics survey showed that the annual economic loss caused due to obesity in the United States is as high as 117 billion US dollars (GBD 2015 Obesity Collaborators et al., 2017). The annual cost of obesity health management in Australia is 30 billion US dollars (Sung et al., 2019). Obesity not only increases the risk of many fatal chronic diseases but also destroys the physical appearance and hinders normal self-recognition and interpersonal communication (GBD 2015 Obesity Collaborators et al., 2017; Kolotkin and Andersen, 2017; Sung et al., 2019). Therefore, it is essential to develop prevention and treatment strategies for overweight and obesity.

Because the pathophysiological mechanisms associated with obesity have not been fully elucidated, current therapies are mostly aimed at improving appetite and reducing weight (Schwartz et al., 2017). Although the existing anti-obesity medications can suppress the strong appetite, they are often accompanied by various adverse effects, such as irritability, paresthesia, insomnia, arrhythmia, and mental illness (Narayanaswami and Dwoskin, 2017; Srivastava and Apovian, 2018; Pilitsi et al., 2019). As a traditional characteristic therapy, electroacupuncture (EA) can regulate appetite and control energy intake without the adverse effects of conventional drugs, thereby making it easy for patients to accept (Lei et al., 2017). Nutritional satisfaction and anorexia are the most pronounced proprioceptive sensations after EA treatment (Wang et al., 2021), but the underlying mechanisms of these phenomena have not been revealed, warranting more extensive research.

The hypothalamus has feeding and satiety centers, which are the primary regulators of the body’s feeding behavior and energy balance (Wang et al., 2021). Autophagy is a ubiquitous homeostatic mechanism in eukaryotes, and in the hypothalamus, it is closely related to the occurrence and development of obesity (Kaushik et al., 2011; Meng and Cai, 2011; Singh, 2012). C57BL/6 mice with long-term high-fat diet (HFD)-induced obesity (DIO) have reduced levels of autophagy-related proteins in the arcuate nucleus of the hypothalamus compared with normal diet mice (Meng and Cai, 2011). The expression of autophagy-related proteins in thalamic GT1-7 cells was increased when cultured under serum-depleted nutrient conditions (Kaushik et al., 2011). Mammalian target of rapamycin (mTOR) is a serine/threonine-protein kinase that senses changes in extracellular nutrients, energy levels, and growth factors (Yang et al., 2012), and tuberous sclerosis complex 1 (TSC1) is an upstream inhibitor of mTOR (Luo et al., 2022). Studies have shown that the methylation rate of the TSC1 promoter in the hypothalamus of obese rats is significantly increased (Wang et al., 2020), and inhibition of this methylation downregulates the expression of mTOR, thereby activating autophagy in the hypothalamus and alleviating the obesity phenotype (Wang et al., 2017; Qi et al., 2019). Therefore, targeting the methylation modification of TSC1-mTOR signaling plays an essential role in regulating autophagy in the hypothalamus.

We previously found that EA at RN12 (*Zhongwan*), ST25 (*Tianshu*), ST36 (*Zusanli*), and SP6 (*Sanyinjiao*) acupoints can downregulate the degree of methylation of the TSC1 promoter in the hypothalamus, inhibit the activities of mTOR, neuropeptide Y (NPY), and Agouti-related protein (AgRP) and increase the expression of pro-opiomelanocortin (PoMC), thereby suppressing appetite and reducing body weight (BW) (Leng et al., 2018). Answers to whether EA can modulate the level of autophagy in the hypothalamus and whether the modulation of EA for hypothalamic autophagy might be related to TSC1-mTOR methylation are yet to be clarified. To address these questions, we employed HFD-induced obese rats as an animal model to investigate the molecular mechanism of the weight-reducing effect of EA and provide the basis for further research in this field.

## Methods

### Animals and grouping

Sixty weans (3-week-old) specific pathogen-free male Wistar rats (weighing 50 ± 10 g) were purchased from Chengdu Dossy Experimental Animals Co., Ltd. (Chengdu, China) and group-housed (four rats per cage) in individually ventilated polyvinylchloride cage systems under a 12/12-h light-dark cycle. After 1 week of acclimation, 50 rats were randomly selected and provided HFD (calorie 4.73 kcal/g; calorie ratio: fat 45 kcal%, carbohydrate 35 kcal%, and protein 20 kcal%. Research Diet, D12451, United States) for inducing obesity, and the remaining 10 rats were fed with a normal diet (calorie 3.85 kcal/g) as the chow group. Average daily food intake of each rat, first calculated as the difference between the amount of food given and the amount of remaining on the next day, then divided by the number of rats in the cage (Liu et al., 2012). Obesity was considered to be induced if the average body weight (BW) was 20% more than of the chow group (Leng et al., 2018).

**FIGURE 1:**
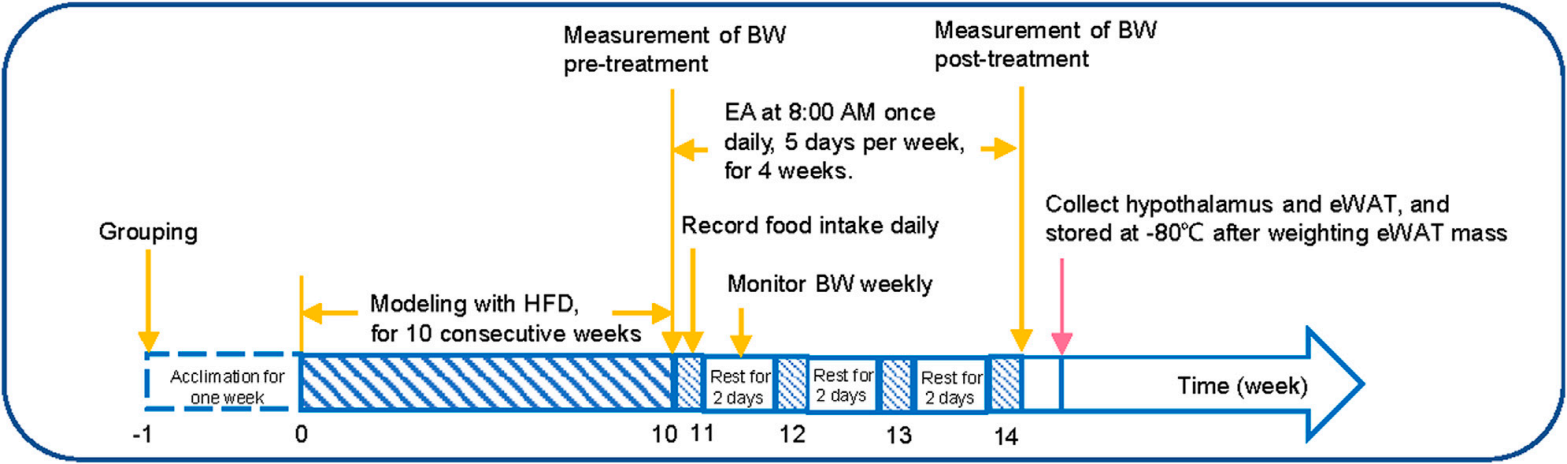
Experimental protocol. (1) week 0–10: the BW of the rats was monitored weekly; (2) week 11–14: the average food intake was recorded daily, and the BW of the rats was recorded on the last day of each week; (3) the day after week 14: the hypothalamus and adipose tissue samples were collected, and the eWAT mass was weighted. HFD, high-fat diet; EA, electroacupuncture; eWAT, epididymal white adipose tissue.

After 10 weeks, 30 successfully modeled rats served as the DIO group (*n* = 10), while others were assigned to either the EA group (*n* = 10) or sham EA group (*n* = 10). All experimental procedures (Figure 1) were strictly according to the National Institutes of Health Guide for the Care and Use of Laboratory Animals and were approved by the Animal Ethics Committee of Chengdu Chinese Medicine University (No. 2019–06). All efforts were made to minimize rats’ suffering.

### Electroacupuncture treatment

From week 11 of the experiment, rats in the EA group were provided EA on RN12 (*Zhongwan*, 20 mm above the umbilicus on the midline of the upper abdomen), ST25 (*Tianshu*, located in the abdomen, the middle side of the umbilicus is opened about 5 mm), ST36 (*Zusanli*, the posterolateral side of the knee joint, about 5 mm under the fibula head), and SP6 (*Sanyinjiao*, the posterior border of the knee joint, about 5 mm above the medial malleolus) after being immobilized in self-made fixators. Sterile acupuncture needles (Huatuo, Suzhou Medical Supplies Co., Ltd. 0.25 gauge ×13 mm) were inserted vertically into the four acupoints at a depth of 3–5 mm and connected to an EA apparatus (HANS-200A, Nanjing, China). The location of acupoints and the depth of penetration were adjusted appropriately according to the degree of obesity. The dilatational waves were set at 2/15 Hz with an intensity level of 1.5 mA for 30 min, once a day, 5 days per week, for a total of 4 weeks.

Rats in the sham EA group were shallowly (transdermal) penetrated at 5 mm away from the acupoints in the EA group, with the electrical stimulation parameters being the same as those for the EA group (Ji et al., 2013). Rats in the chow and DIO groups were restrained in the same way without administering any treatment. The BW of the rats was monitored weekly. After 4 weeks of EA treatment, all rats were anesthetized with pentobarbital sodium (40 mg/kg), and hypothalamus and adipose tissue samples were collected.

### Morphological analysis of white adipose tissue

The epididymal white adipose tissues (eWATs) were fixed with 4% paraformaldehyde, dehydrated, embedded in paraffin, and sliced into 4 µm thick sections. These sections were then stained with hematoxylin and eosin (H&E). After staining, images of five randomly selected sections of each group were dehydrated and photographed using an optical microscope (Olympus, Japan). Finally, adipocyte area (µm^2^) and diameter (µm) were averaged from 10 cells from five tissue sections for each sample with Image-Pro Plus software (Media Cybernetics, Version 6.0), and the methods previously described by Parlee SD et al., in 2014 (Parlee et al., 2014). The software was operated by an investigator blinded to the group assignment.

### Immunofluorescence analysis

The hypothalamus tissues were fixed with 4% paraformaldehyde, dehydrated, embedded, and sliced, immersed in 0.01 M citrate buffer (pH 8.0), heated until boiling, washed with PBS, blocked with 10% serum for 30 min. Primary antibodies for LC3 (3868T, 1:200, CST) were dropped into the samples. After the samples were washed with PBS, a secondary antibody (GB21303, 1:200, Servicebio) and DAPI were dropped, and the samples were incubated at room temperature for 10 min, washed with PBS, and sealed with fluorescence decay-resistant sealing tablets.

### Western blot analysis

Measurement of autophagy components (Beclin-1, LC3-Ⅰ/LC3-Ⅱ, and p62) and mTOR signaling (mTOR, p-mTOR, p70S6K, p-p70S6K) protein expressions in the hypothalamus by WB. Total protein concentration was measured with the BCA protein quantification kit (Beyotime, China). Samples were separated by electrophoresis, transferred to a PVDF membrane (HyBond, United States), and placed in 5% skimmed milk. The protein bands were incubated with the following primary antibodies: Beclin-1 (ab207612, 1:2000, Abcam), LC3B (3868S, 1:1000, CST), SQSTM1/p62 (ab109012, 1:10000, Abcam), mTOR (ab134903, 1:10000, Abcam), p-mTOR (5536S, 1:1000, CST), p70S6K (ab32529, 1:5000, Abcam), and p-p70S6K (9234S, 1:1000, CST). The membrane was incubated with the secondary antibodies anti-mouse IgG (H + L) (A0208, 1:1000, Beyotime) or anti-rabbit IgG (H + L) (A0216, 1:1000, Beyotime) after washing. Relative expressions of autophagy-related components and mTOR signaling were obtained by normalization against GAPDH levels.

### Bisulfite genomic sequence analysis

After extracting DNA by the DNA Extraction Kit (Tiangen, China), 1.0 μg of DNA from each sample was treated with bisulfite, using a DNA methylation kit (QIAGEN, Germany). PCR amplification of the bisulfite-treated DNA was done. The TSC1 promoter primers sequences included F: 5′-GAT​TGT​TGT​TAA​TAA​TAA​TGT​GAT​GTG -3′ and R: 5′-TCA​CCA​CAA​CTA​CTC​CTA​CTC​AAC -3′. Amplification was carried out by denaturing at 95°C for 10 min, followed by 35 cycles of 94°C for 30 s, 55°C for 30 s, 72°C for 30 s, and finally 72°C for 5 min. PCR purified products were connected to pTG19-T vector and transformed into competent cells and then positive clones and extract plasmids were selected. We judged whether the 50-C-phosphate-G-30 (CpG) positions were methylated referring to the principle that sodium bisulfite cannot convert thymidine to methylation.

### Statistical analysis

Normally and nonnormally distributed data were expressed as Mean ± standard deviation (SD) and median (IQR), and analyzed by SPSS 22.0 software (IBM Statistics, Armonk, NY, United States). After the data of each group met the normal test and homogeneity analysis of variance, ANOVA was used for comparison between groups. If homogeneity of variance and the normality assumption were violated, a non-parametric test (Kruskal–Wallis) was used. Results were considered significant when the *p* < 0.05.

## Results

### Electroacupuncture treatment markedly decreased BW and adipose tissue mass in high-fat diet-fed rats

**FIGURE 2:**
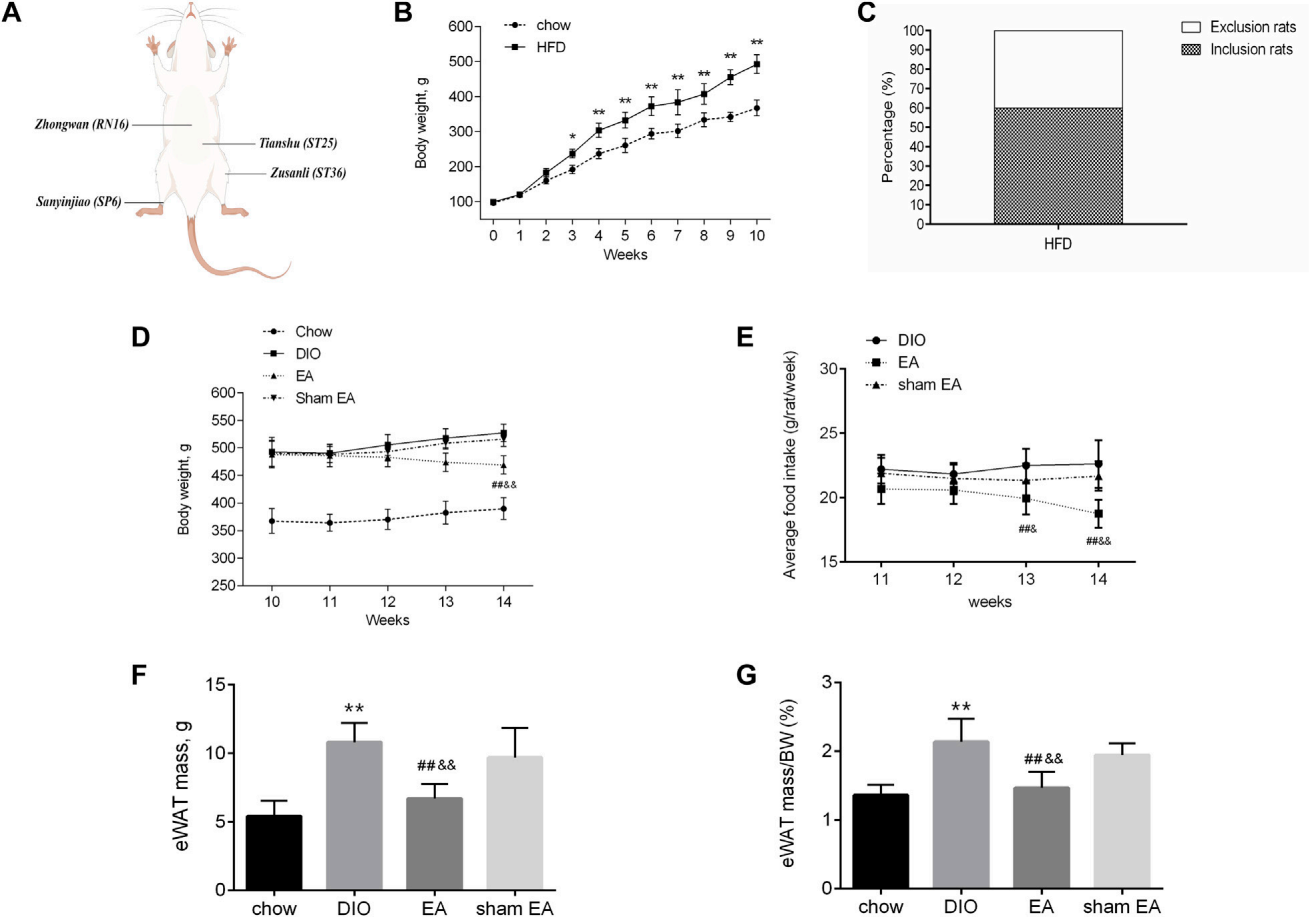
EA treatment decreased body weight and adipose tissue mass in the HFD-fed rats. **(A)** The location of prescribed acupoints, **(B)** BW from the chow and HFD groups from week 0 to week 10, **(C)** proportion of rats gained 20% or more than the average BW of the chow group, **(D)** BW from the four groups within week 10 to week 14, **(E)** average food consumption per rat within week 11 to week 14, **(F)** eWAT mass, and **(G)** eWAT/BW ratio from the four groups. All data are expressed as Mean ± SD (*n* = 10 rats per group), **p < 0.01 vs. chow; ^##^ *p* < 0.01 vs. DIO; ^&&^ *p* < 0.01 vs. sham EA. HFD, high-fat diet; EA, electroacupuncture; DIO, diet-induced obesity; BW, body weight; eWAT, epididymal white adipose tissue. EA treatment reversed HFD-induced morphological alternations.

After 10 weeks of consuming HFD, the BW of the HFD group increased significantly compared to the chow group (Figure 2B), in which 30 rats (60.00%) gained 20% or more BW compared with the average BW of the chow group (Figure 2C) and were therefore randomized into DIO, EA, and sham EA groups. Treatment with EA significantly reduced the BW and food consumption of rats in the EA group compared to rats in the DIO and sham EA groups (Figures 2D,E). Obesity is associated with dynamic adipose tissue remodeling characterized by adipocyte hypertrophy, especially in the epididymal white adipose tissue (eWAT). As shown in Figures 2F,G, eWAT mass and eWAT/BW ratio were increased in obese rats, but EA treatment attenuated these increases. The eWAT mass and eWAT/BW ratio did not significantly differ between the DIO and sham EA groups (Figures 2F,G).

**FIGURE 3:**
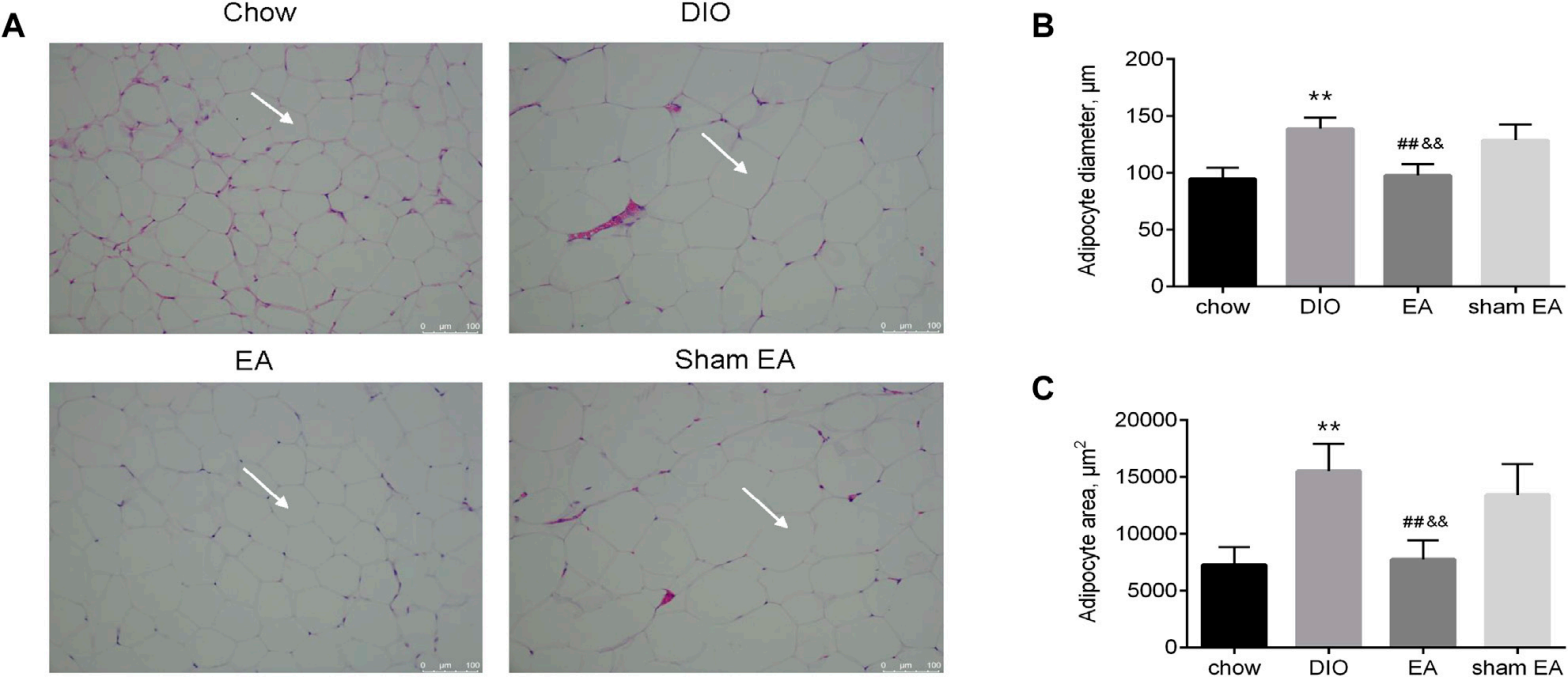
EA treatment reversed HFD-induced pathological morphological changes in the eWAT. **(A)** Representative H&E (×200) staining sections (scale bar, 100 μm). The white arrows indicate adipocyte, **(B)** adipocyte diameter, and **(C)** adipocyte area. All data are expressed as Mean ± SD (*n* = 6 rats per group), **p < 0.01 vs. chow; ^##^ *p* < 0.01 vs. DIO; ^&&^ *p* < 0.01 vs. sham EA. HFD, high-fat diet; eWAT, epididymal white adipose tissue; EA, electroacupuncture; DIO, diet-induced obesity. EA treatment modulated the expression of autophagy-related components in the hypothalamus.

H&E staining was performed to detect morphological changes in the eWAT (Figure 3A). Adipocytes in the DIO group were larger than those in the chow group; but EA treatment decreased the diameter and area of adipocytes compared to the DIO and sham EA groups (Figures 3B,C). Consistent with results supporting the weight reduction effect of EA, the adipocyte size was not altered in the sham EA group compared to the DIO group.

**FIGURE 4:**
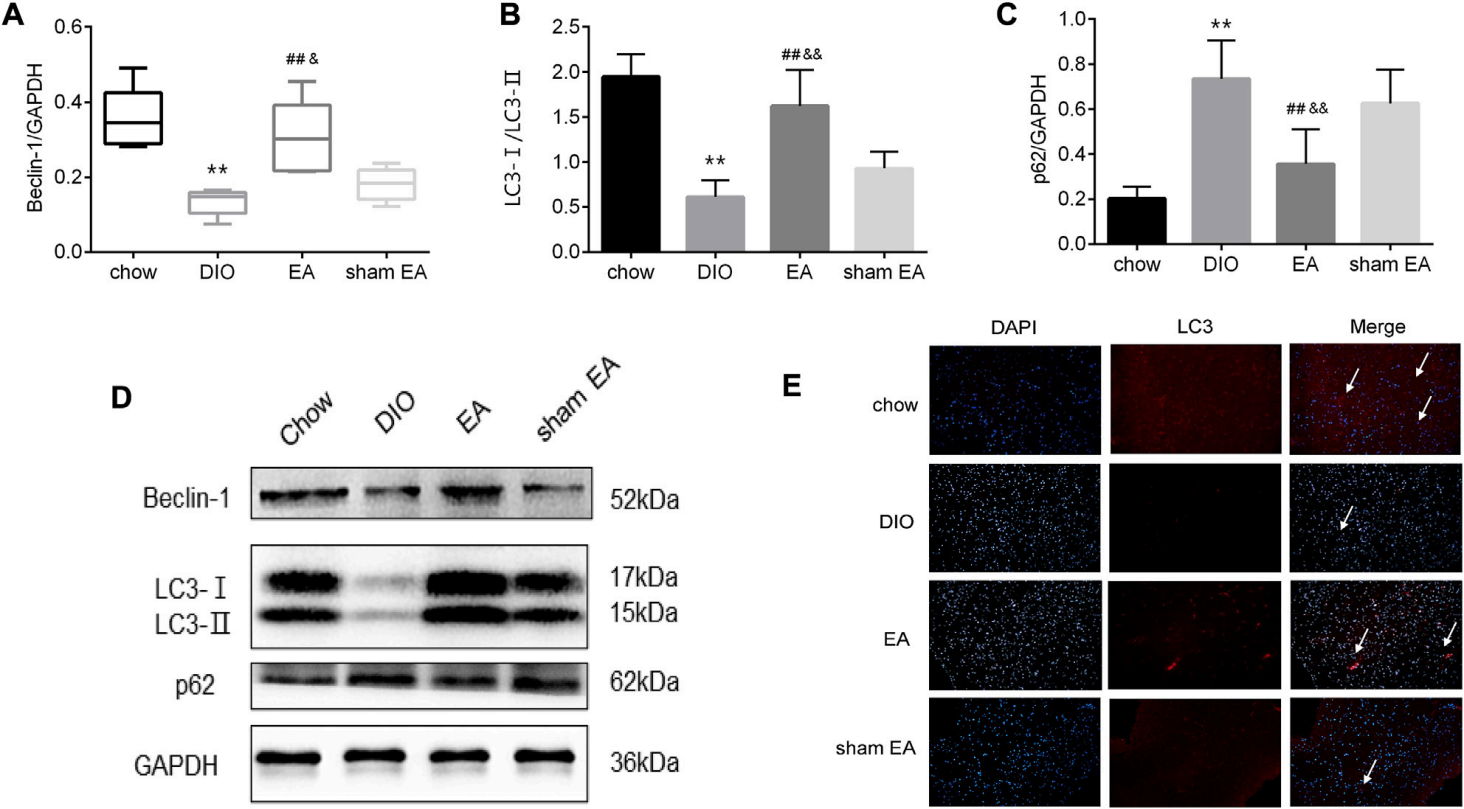
EA treatment alters the expression of autophagy-related biomarkers in the hypothalamus. **(A)** Relative protein expressions of Beclin-1, **(B)** LC3-Ⅰ/LC3-Ⅱ, and **(C)** p62 detected by Western Blot; **(D)** immunoblot image showing Beclin-1, LC3-Ⅰ/LC3-Ⅱ, and p62; **(E)** representative images showing DAPI (blue) and LC3 (red) fluorescence (scale bar, 100 μm). The white arrows in the merged images indicate LC3. Data are expressed as Mean ± SD or Median (IQR) (*n* = 2 rats per group for IF, and *n* = 5 rats per group for others), **p < 0.01 vs. chow; ^##^ *p* < 0.01 vs. DIO; ^&&^ *p* < 0.01 vs. sham EA. EA, electroacupuncture; DIO, diet-induced obesity; IF, immunofluorescence. EA treatment decreased mTOR and p70S6K protein expression in the hypothalamus.

Given that autophagy in the hypothalamus has been identified to regulate obesity, we then assessed the effect of EA on autophagy-related components LC3, p62, and Beclin-1. Western blot analysis showed that rats in the DIO group had lower protein expressions of Beclin-1 and LC3-Ⅱ/LC3-Ⅰ ratio, and higher protein expression of p62 than rats in the chow group (Figures 4A–D). EA treatment normalized these changes in the DIO group by increasing Beclin-1 expressions and LC3-Ⅱ/LC3-Ⅰ ratio, and reducing p62 expressions (Figures 4A–D). In contrast, there were no differences in expressions of Beclin-1, LC3-Ⅱ/LC3-Ⅰ, and p62 between the DIO group and the sham EA group. Moreover, in EA-treated rats, LC3-immunoreactive nuclei accumulated in the hypothalamus (Figure 4E). These findings indicate that EA treatment may reduce BW by modulating the expression of autophagy-related components in the hypothalamus.

**FIGURE 5:**
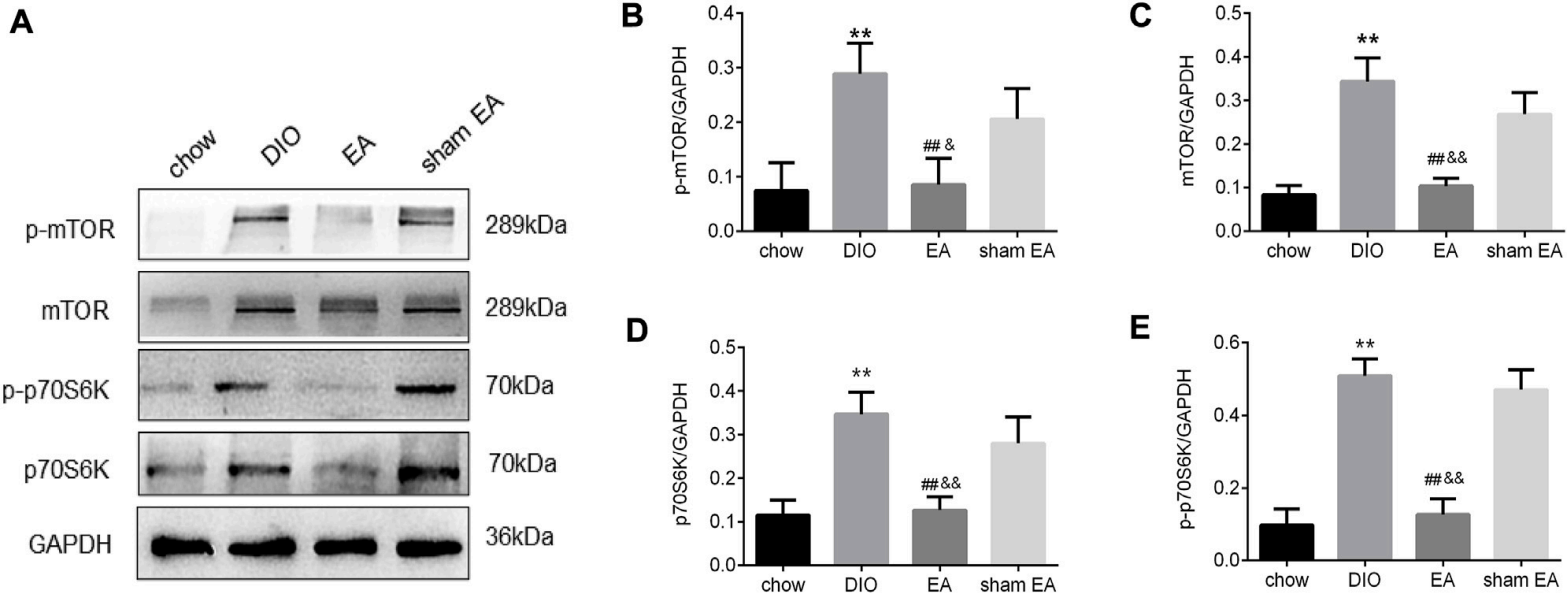
EA treatment suppresses the protein expression of mTOR and p70S6K in the hypothalamus. **(A)** Immunoblot image showing mTOR, p-mTOR, p70S6K, and p- p70S6K; **(B)** relative protein expressions of p-mTOR, **(C)** mTOR, **(D)** p70S6K, and **(E)** p- p70S6K detected by Western Blot. All data are expressed as Mean ± SD (*n* = 5 rats per group), **p < 0.01 vs. chow; ^##^ *p* < 0.01 vs. DIO; ^&&^ *p* < 0.01 vs. sham EA. EA, electroacupuncture; DIO, diet-induced obesity. EA treatment decreased the methylation rate of the TSC1 gene promoter in the hypothalamus.

Next, we further investigated the expression levels of mTOR and its downstream protein p70S6K to illustrate the potential mechanism of EA-induced changes in hypothalamic autophagy. As shown in Figures 5A–E, the continuous consumption of HFD significantly upregulated the levels of mTOR, p-mTOR, p70S6K, and p-p70S6K. The levels of mTOR, p-mTOR, p70S6K, and p-p70S6K were significantly lower in the EA group than in the DIO and sham EA groups (Figures 5A–E). Again, there were no differences in these parameters between the sham EA group and the DIO group. Moreover, no clear trend was found in ratios of p-mTOR/mTOR and p-p70S6K/p70S6K, suggest that EA might modulate total mTOR protein levels more than phosphorylation levels (Supplementary Figures S1A,B).

**FIGURE 6:**
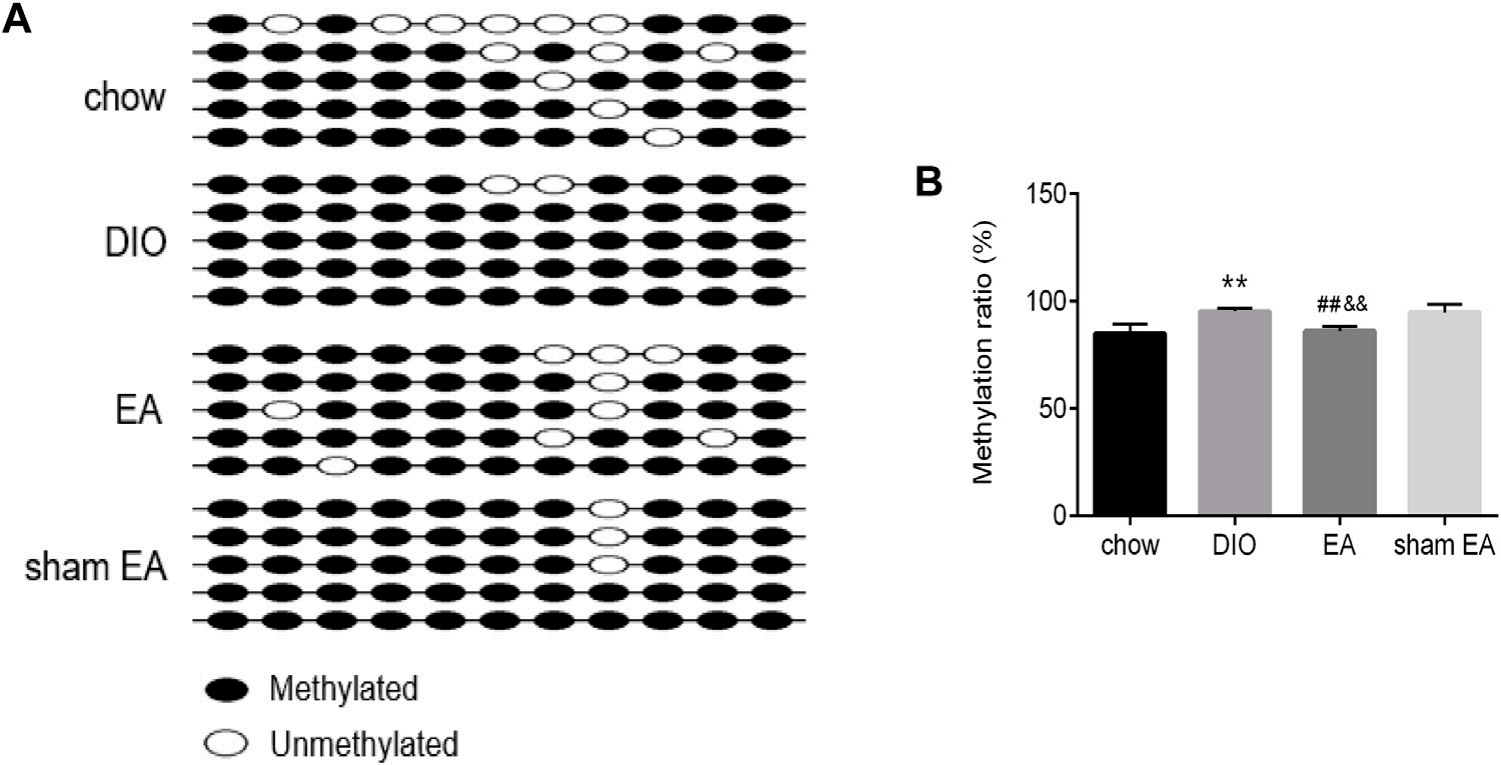
EA treatment decreased the methylation rate of *TSC1* gene promoter in the hypothalamus. **(A)** Lollipop-style representation of methylated/unmethylated sites of *TSC1* gene promoter; **(B)** the methylation ratio of *TSC1* gene promoter detected by BSP analysis. All data are expressed as Mean ± SD (*n* = 5 rats per group), **p < 0.01 vs. chow; ^##^ *p* < 0.01 vs. DIO; ^&&^ *p* < 0.01 vs. sham EA. BSP, Bisulfite Genomic Sequence; EA, electroacupuncture; DIO, diet-induced obesity.

BSP analysis was performed to test the methylation level of the *TSC1* gene promoter in the rat hypothalamus. The sequenced fragment contained 11 CpG loci (Figure 6A). According to BSP data (Figures 6A,B), rats in the DIO group had a significantly higher methylation rate of *TSC1* gene promoter than rats in the chow group. After EA treatment, the methylation rate of the *TSC1* gene promoter was significantly downregulated compared to the DIO and sham EA groups. There was no difference in the methylation level of the *TSC1* gene promoter between the sham EA group and the DIO group (Figures 6A,B).

## Discussion

### Electroacupuncture treatment could effectively reduce the body weight and fat accumulation in DIO rats

Due to the current lifestyle and sedentary work patterns, obesity has become widespread worldwide. Based on complex network technology, the results of prescription analysis of EA treatment for obesity have shown that RN12 (*zhongwan*), ST25 (*tianshu*), ST36 (*zusanli*), and SP6 (*sanyinjiao*) are the top four acupoints with the highest core degree (Chen et al., 2018). The results of our previous randomized controlled trial on obese individuals showed that EA at RN12, ST25, ST36, and SP6 acupoints could significantly reduce BW, waist-to-hip ratio, and body fat percentage (Zhang et al., 2012). In our animal study following this treatment protocol, EA significantly reduced BW and abdominal fat content in DIO rats, confirming the weight-reducing effect of EA (Leng et al., 2018). In the present study, EA could effectively reduce the body weight and epididymal white fat mass of DIO rats and the diameter and area of epididymal adipocytes, suggesting the fat-reducing effect of EA, in line with previous research results on cell morphology.

Secondly, our study confirmed that the selection of acupoints is one of the key factors affecting the efficacy of EA. Some studies have reported that electrical conductivity is higher at meridian and acupoints than at non-meridian and non-acupoints, and the meridian system may be the conduit through which the current passes (Jia et al., 2012; Yao et al., 2022). Research on acupoints of the spleen and stomach meridian in experimental rats showed that the nerve density of acupoints was related to the strength of electric supply (Ma et al., 2012). The higher the acupoint voltage, the higher the nerve density, i.e., the nerve density of non-acupoints was significantly lower than that of acupoints (Ma et al., 2012). To verify the pure effect of the selected acupoints on obesity treatment, we designed a group of rats treated with sham EA at non-acupoints. The findings showed that the sham EA treatment did not significantly reduce BW and the diameter and area of adipocytes, suggesting that EA therapy is not merely physical stimulation but also involves specific acupoint effects. The results of this study are in line with the conclusions of our previous systematic review (Yao et al., 2019).

### Electroacupuncture treatment for weight reduction may be closely related to the regulation of the hypothalamic autophagy

The process of autophagy allows intracytoplasmic ingredients to be degraded at lysosomes for energy production. In the early stage of autophagosome formation, Beclin-1 binds to Vps34 to form a type III PI3K complex and then, together with Atg1 and Atg9, regulates phagosome formation (Song et al., 2021). LC3 is cleaved by Atg4 protease to form LC3-I, which then couples with phosphatidylethanolamine to form LC3-II, which attaches to the surface of the autophagic vesicle (Dong et al., 2022). p62 can bind to ubiquitin substrates and LC3, which are integrated into autophagosomes by binding to LC3-II and finally degrade autophagic lysosomes (Jeon et al., 2021).

The levels of hypothalamic autophagy are closely related to the regulation of neuropeptides, as well as the appetite and feeding behaviors. Agouti-related protein (AgRP) and Pro-opiomelanocortin (POMC), the primary neuropeptides in the hypothalamic, have opposite functions in the regulation of appetite (Vohra et al., 2022). Autophagy in AgRP neurons controls food intake and energy balance by modulating AgRP levels, whereas the loss of autophagy in POMC neurons affected hypothalamic anorexic peptides to promote adiposity (Bouret, 2022). Studies in AgRP-expressing hypothalamic GT1-7 cells revealed that starvation-induced autophagy mobilized hypothalamic lipids to generate endogenous free fatty acids that increased AgRP levels, thus revealing the role of hypothalamic autophagy in regulating orexigenic AgRP (Kaushik et al., 2011). In response to starvation, the physiologically increased expression of the neurotransmitter is reduced, which upregulates POMC and α-MSH, resulting in a lean phenotype in the mice (Coupé et al., 2012). By constructing gene knockout animal models, the researchers re-examined the relationship between hypothalamic autophagy and obesity. The accumulation of p62 protein can be induced by POMC in neurons lacking ATG7 (Kaushik et al., 2012). Atg7-knockout mice had a better appetite, consumed lesser energy, and gained more weight than control mice (Kaushik et al., 2012). All the above studies have shown that changes in autophagy in the hypothalamic are related to feeding and energy regulation, and regulation of autophagy may be a potential mechanism for reducing weight. Our results showed that the expression of Beclin-1 and LC3-II/LC3-I ratio decreased and the expression of p62 protein increased in the hypothalamus of DIO model rats, while EA effectively upregulated the expression of Beclin-1 and LC3-II/LC3-I ratio and downregulated the expression of p62, consistent with the abovementioned reports.

### Electroacupuncture treatment may regulate the hypothalamic autophagy by demethylating tuberous sclerosis complex 1-mammalian target of rapamycin signaling

Methylation modification of the TSC1-mTOR plays an important role in the transduction of autophagy signaling (Qi et al., 2019; Bjune et al., 2022). Methylation, an important epigenetic regulatory mechanism, refers to the transfer of methyl groups to specific bases on DNA molecules by using S-adenosylmethionine as a methyl donor under the catalysis of DNA methyltransferase (Bjune et al., 2022). One of the protein products of the *TSC* gene is TSC1, which has GTPase activity and is an important inhibitory regulator upstream of the mTOR signaling pathway (Wang et al., 2020; Luo et al., 2022). In the obese state, the methylation level of the *TSC1* gene promoter region increases, and inhibition of downstream mTOR is abolished (Wang et al., 2020). mTOR then phosphorylates the effector p70S6 kinase (p70S6K), which in turn inhibits a series of downstream signal transduction cascades formed by ATG complexes to regulate autophagy (Jin et al., 2018; Zhao et al., 2021). We have previously shown that methylation of the promoter of the *TSC1* gene in the hypothalamus of HFD-fed obese rats is increased and the inhibition of *mTOR* gene expression is weakened (Wang et al., 2020), and EA can demethylate TSC1 in the mTOR signaling pathway (Leng et al., 2018; Wang et al., 2020). However, the downstream targets of mTOR that EA against obesity and the underlying mechanism are not been examined. Kaushik S et al. found that the expression of LC3-II was increased, and mTOR phosphorylation and the activity of substrate p70S6K were decreased in hypothalamic GT1-7 cells cultured under serum-starved conditions (Kaushik et al., 2011). When GT1-7 cells were re-cultured with serum, LC3-II levels decreased, and the activity of mTOR and p70S6K were increased (Kaushik et al., 2011; Oh et al., 2016). Our current study is the advanced research of the previous one, we improved the methylation detection technique to BSP and detected the phosphorylation levels of mTOR and p70S6K protein additionally. The results also showed that EA could downregulate the methylation rate of the *TSC1* gene and the expression level of mTOR and p70S6K. Importantly, we further investigate the possible downstream targets of mTOR that EA against obesity. The results found that EA could regulate the level of hypothalamic autophagy, which in turn reduces food consumption and body weight. Collectively, the objectives of the two studies are continuous, thus ensuring a more comprehensive disclosure of the mechanism of EA for weight loss.

In summary, the present study confirms that EA induces weight loss, at least in part, due to the activation of autophagy in the hypothalamus by demethylating the TSC1-mTOR signaling pathway, thereby inhibiting fat accumulation. Our study also existed some limitations. Firstly, the autophagy signaling in the hypothalamus was not blocked in this study. In the future, we will use autophagy inhibitors or inducers to clarify further the causal relationship between the weight reduction effect of EA and changes in hypothalamic autophagy of DIO rats. In addition, we will block the methylation of TSC promoter to verify whether the methylation of TSC1/mTOR is a key mechanism by which EA regulates autophagy in the hypothalamus.

## Conclusion

EA could reduce body weight and fat accumulation in DIO rats. This ameliorative effect of EA may be associated with its demethylation effect on TSC1-mTOR and regulation of hypothalamic autophagy. In the future, we will use biochemical methods to reduce protein content or block the pathway in order to reverse the effect of autophagy or EA. Our results will provide the basis for promoting the utilization of EA in the clinical scenario for obese individuals.

## Data Availability

The original contributions presented in the study are included in the article/Supplementary Material, further inquiries can be directed to the corresponding author.
