# Supplementary material for: Electroacupuncture induces weight loss by regulating tuberous sclerosis complex 1-mammalian target of rapamycin methylation and hypothalamic autophagy in high-fat diet-induced obese rats
Source: Front Pharmacol. 2022 Oct 12;13:1015784. doi: 10.3389/fphar.2022.1015784 (PMC9596966; doi:10.3389/fphar.2022.1015784)
Supplement: Supplementary file 1 [file DataSheet3.docx]

Supplementary Material

# Supplementary Data

Although significant differences were shown in p-mTOR, mTOR, p-p70S6K, and p70S6K, no clear trend was found in ratios of p-mTOR/mTOR and p-p70S6K/p70S6K, suggest that EA might modulate total mTOR protein levels more than phosphorylation levels (**Supplementary** **Figure 1A-B**).

# Supplementary Figures

**A**





**B**





**Supplementary Figure 1 ratios of p-mTOR/mTOR and p-p70S6K/ p70S6K** (A) p-mTOR/mTOR and (B) p-p70S6K/p70S6K. The data are expressed as mean ± SD (n=5 rats per group). Ns represents *P*＞0.05 between marked groups.
